# Characteristics and Outcomes of Intravenous Thrombolysis in Mild Ischemic Stroke Patients

**DOI:** 10.3389/fneur.2021.744909

**Published:** 2021-10-29

**Authors:** Huan Tang, Shenqiang Yan, Chenglong Wu, Yanxing Zhang

**Affiliations:** ^1^Department of Neurology, Shaoxing People's Hospital (Shaoxing Hospital, Zhejiang University School of Medicine), Shaoxing, China; ^2^Department of Neurology, Second Affiliated Hospital of Zhejiang University School of Medicine, Hangzhou, China

**Keywords:** mild ischemic stroke, thrombolysis, early neurological deterioration, unfavorable outcome, systolic blood pressure (SBP)

## Abstract

**Objective:** This study assessed the characteristics of intravenous thrombolysis (IVT) with respect to early neurological deterioration (END) and functional outcome in mild ischemic stroke patients.

**Methods:** Data were obtained from acute mild ischemic stroke patients (defined as having a National Institute of Health Stroke Score (NIHSS) ≤ 5) treated with IVT in our hospital from July 2017 to December 2020. END was defined as the NIHSS increased ≥1 over the baseline at 24 h after IVT. A modified Rankin scale (mRS) ≤ 1 at 3 months was considered as a favorable outcome, and an mRS ≥2 at 3 months was an unfavorable outcome.

**Results:** Two hundred thirty-three acute mild ischemic stroke patients (all patients underwent MRI and DWI restriction) with IVT were included in this study. Thirty-one patients experienced END, and 57 patients experienced an unfavorable outcome at 3 months. With multivariate analysis, END was associated with an elevated baseline systolic blood pressure (SBP) (OR = 1.324, 95% CI, 1.053–1.664, *p* = 0.016) and coronary heart disease (OR = 4.933, 95% CI, 1.249–19.482, *p* = 0.023). An unfavorable outcome at 3 months after IVT was independently associated with a baseline elevated SBP (OR = 1.213, 95% CI, 1.005–1.465, *p* = 0.045), baseline NIHSS (OR = 1.515, 95% CI, 1.186–1.935, *p* = 0.001), prior hyperlipemia (OR = 3.065, 95% CI, 1.107–8.482, *p* = 0.031), cardioembolic stroke (OR = 0.323, 95% CI, 0.120–0.871, *p* = 0.025), and END at 24 h (OR = 4.531, 95% CI, 1.950–10.533, *p* < 0.001) in mild ischemic stroke patients.

**Conclusion:** In mild ischemic stroke patients with IVT, an elevated baseline SBP and coronary heart disease were associated with END. The elevated baseline SBP, baseline NIHSS, a history of prior hyperlipemia, cardioembolic stroke, and END at 24 h after IVT were useful in predicting an unfavorable outcome at 3 months.

## Introduction

Intravenous thrombolysis (IVT) has been proven to be an effective treatment for acute ischemic stroke patients when it is given within 4.5 h of stroke onset (1). More than half of acute ischemic stroke patients exhibit mild symptoms, including neurological deficits (2, 3). Currently, mild ischemic stroke has no uniform definition, but most studies define mild ischemic stroke as presenting a National Institute of Health Stroke Score (NIHSS) ≤ 5 (4, 5). Due to the risk of hemorrhagic transformation and not increasing the likelihood of a favorable outcome at 90 days (5), IVT is not recommended for mild non-disabled ischemic stroke patients within 4.5 h (6). However, approximately one-third of mild ischemic stroke patients without IVT have unfavorable outcome due to mild stroke (7). Some non-disabled patients who did not receive IVT in the appropriate time frame go on to develop early neurological deterioration (END) (8). Currently, there are no better-accepted treatments that can be given to lengthen the time window of IVT. However, previous research has found that mild ischemic stroke patients could be benefit from IVT (9–11). Although thrombolysis might increase the risk of hemorrhagic transformation, but its occurrence was statically insignificant and did not increase the mortality rate (9, 11). Therefore, this study was designed to identify factors that impacted END and the functional outcome of mild ischemic stroke patients after IVT, which could be useful in predicting a possible unfavorable outcome at 3 months.

## Methods

### Patient Selection

The study was carried out between July 2017 and December 2020 in Shaoxing People's Hospital (Shaoxing Hospital, Zhejiang University School of Medicine). Initially, there were 282 acute, mild, and ischemic stroke patients receiving intravenous recombinant tissue plasminogen activator (alteplase 0.9 mg/kg up to a maximum of 90 mg, 10% of the total dosage as a bolus and the rest over 1 h) therapy in our hospital. We eliminated 49 patients (25 patients were stroke mimic, 2 patients were newly diagnosed lung cancer, 18 patients lacked follow-up data, and 4 patients lacked MRI images). Finally, 233 patients were enrolled in this study. Patients were selected using the following criteria: (1) is aged >18 years; (2) diagnosed with acute ischemic stroke (AIS) according to clinical symptoms, MRI, and DWI restriction; (3) had a baseline NIHSS ≤ 5; (4) received IVT within 4.5 h of AIS onset; and (5) underwent CT scans at 24 h after IVT. The exclusion criteria included the following: (1) the presence of existing contraindications for intravenous thrombolysis according to the standard IVT guidelines (12); (2) prior stroke or the presence of other diseases that resulted in a baseline mRS ≥1; (3) a long-term life expectancy of 3 months or less; (4) necessity of daily life need care because of other chronic system diseases, such as chronic heart failure, chronic obstructive pulmonary disease, and end-stage renal disease; (5) limb fracture affecting movement; and (6) lack of follow-up data.

### Data Collection

A neurologist who was blinded to the patient's outcome reviewed the medical records to collect the following data: demographic characteristics, baseline NIHSS, baseline SBP, baseline DBP, history of smoking, hypertension, atrial fibrillation, coronary heart disease, diabetes, prior hyperlipemia, and prior stroke or transient ischemic attack (TIA). A history of prior hyperlipemia disease included hypertriglyceridemia and hypercholesterolemia. Coronary heart disease is defined as a patient having a history of acute coronary syndrome or angina pectoralis. Acute ischemic stroke subtypes were determined by using the TOAST (Trial of Org 10172 in Acute Stroke Treatment) classification (13). This study was approved by the Shaoxing Hospital, Zhejiang University School of Medicine Sciences Ethics Committee.

### Neurological Outcomes

END was defined as an NIHSS at 24 h after IVT that was increased ≥1 over the baseline (14). There was a neurologist in charge of the follow-up. The mRS score at 3 months was all collected by phone. An mRS score ≤ 1 at 3 months was determined to be a favorable outcome after IVT, and an mRS score ≥2 at 3 months was an unfavorable outcome. Hemorrhage transformation was classified as hemorrhagic infarction types I and II and parenchymal hemorrhage types I and II according to the definition provided by the European Cooperative Acute Stroke Study (ECASS) (15). Symptomatic intracerebral hemorrhage (SICH) was defined as the presence of a neurological decline attributed to parenchymal hemorrhage type II with an NIHSS score increase of ≥4 after IVT (16).

### Statistical Analysis

Statistical analysis was performed using SPSS version 17.0 (SPSS Inc., Chicago, Illinois, USA). Fisher's exact test was used to compare the dichotomous variables between groups, while the Mann–Whitney *U*-test was used for the continuous variables. Variables with a two-tailed *p*-value of < 0.1 in univariate regression analyses were included in the binary multivariate logistic regression model to determine the independent risk factors of END at 24 h and functional outcome at 3 months after IVT. A two-tailed *p*-value < 0.05 was considered statistically significant.

## Results

### Baseline Characteristics

Two hundred thirty-three mild AIS with IVT were included. No patients underwent thrombectomy. The mean age (±SD) was 60.08 ± 10.87 years. Eighty-four (36.1%) patients were female. The median baseline NIHSS was 2 (interquartile range, IQR, 1–4) among all patients: 31 (13.3%) patients experienced END, 9 (3.9%) patients experienced hemorrhagic transformation, and 2 (0.9%) of the nine exhibited SICH. Nine patients experienced stroke recurrence in 3 months. Fifty-seven (24.5%) patients experienced an unfavorable outcome at 3 months after IVT. The demographics and baseline characteristics are shown in Table 1.

**Table 1:** Characteristics of mild acute ischemic stroke patients with IVT with or without END at 24 h.

	**END at 24 h** ***N* = 31**	**Non-END** **at 24 h** ***N* = 202**	***p*-value**
Age, IQR	68 (63–73)	67 (58–74)	0.979
Female, *N* (%)	14 (45.2)	70 (34.7)	0.315
ONT, (mean ± SD)	183.65 ± 66.64	178.60 ± 81.91	0.541
Baseline NIHSS, IQR	3 (2–5)	3 (2–4)	0.246
Baseline SBP, mmHg	157.19 ± 18.54	148.47 ± 18.15	**0.007**
Baseline DBP, mmHg	87.74 ± 8.53	84.28 ± 11.45	0.059
Smoking, *N* (%)	8 (26.7)	53 (27.3)	1.000
Hypertension, *N* (%)	22 (71)	138 (68.3)	0.838
Atrial fibrillation, *N* (%)	6 (19.4)	29 (14.4)	0.429
Coronary heart disease, *N* (%)	4 (12.9)	6 (3.0)	**0.031**
Diabetes, *N* (%)	7 (22.6)	45 (22.3)	1.000
Hyperlipemia, *N* (%)	2 (6.5)	20 (9.9)	0.747
Prior stroke or TIA, *N* (%)	4 (12.9)	36 (17.9)	0.615
TOAST criteria			
Large artery atherosclerosis, *N* (%)	11 (35.5)	52 (26.2)	0.286
Cardioembolic, *N* (%)	6 (19.4)	42 (20.8)	1.000
Lacunar, *N* (%)	9 (29.0)	70 (34.7)	0.684
Undetermined cause, *N* (%)	4 (12.9)	34 (16.8)	0.795
Other etiology, *N* (%)	1 (3.2)	3 (1.5)	0.437
Hemorrhagic transformation, *N* (%)	4(12.9)	5(2.5)	**0.020**
HI-I, *N* (%)	1 (3.2)	4 (2.0)	
HI-II, *N* (%)	0 (0)	1 (0.5)	
PH-I, *N* (%)	1 (3.2)	0 (0)	
PH-II, *N* (%)	2 (6.5)	0 (0)	
Symptomatic intracerebral hemorrhage, *N* (%)	2 (6.5)	0 (0)	**0.017**
3 mRS ≥ 2, *N* (%)	17 (54.8)	40 (19.8)	** < 0.001**

*SBP, systolic blood pressure; DBP, diastolic blood pressure; HI-I, hemorrhagic infarction type; HI-II, hemorrhagic infarction type II; PH-I, parenchymal hemorrhage type I; PH-II, parenchymal hemorrhage type II*.

### Early Neurological Deterioration

After univariate analysis is carried out, the mild AIS patients with END exhibited a higher baseline SBP (157.19 ± 18.54 vs. 148.47 ± 18.15, *p* = 0.007) and a higher baseline DBP (87.74 ± 8.53 vs. 84.28 ± 11.45, *p* = 0.059) compared with non-END patients. Moreover, the mild AIS patients with END presented a higher rate of coronary heart disease compared with the non-END group (12.9% vs. 3.0%, *p* = 0.031). Baseline SBP, DBP, and coronary heart disease were included in the binary logistic multivariate analysis. The results revealed that an elevated baseline SBP (OR = 1.324, 95% CI, 1.053–1.664, *p* = 0.016) and coronary heart disease (OR = 4.933, 95% CI, 1.249–19.482, *p* = 0.023) were independently associated with END at 24 h after IVT (Table 2).

**Table 2:** Binary logistic analysis of risk factors of END at 24 h after IVT.

	**OR**	**95% CI**	***p***
Baseline SBP	1.324	1.053–1.664	**0.016**
Baseline DBP	1.107	0.727–1.684	0.636
Coronary heart disease	4.933	1.249–19.482	**0.023**

### Neurologic Outcome

In this study, 57 (24.5%) mild AIS patients experienced an unfavorable outcome. Two patients experienced SICH and had an unfavorable outcome at 3 months. Based on univariate analysis, we observed that patients with unfavorable outcomes at 3 months had a higher baseline NIHSS (IQR, 3 (2–4) vs. 3 (2–5), *p* = 0.001), a higher baseline SBP (154.68 ± 17.27 vs. 147.99 ± 18.51, *p* = 0.016), and increased END (29.8% vs. 8.0%, *p* < 0.001) after IVT. In the TOAST classification, the unfavorable outcome group had a higher rate of large artery atherosclerosis (40.4% vs. 23.3%, *p* = 0.017) and a lower rate of cardioembolic stroke (10.5 vs. 23.9%, *p* = 0.037). However, there was no difference in the incidence of hemorrhagic transformation (5.3 vs. 3.4%, *p* = 0.692) between the two groups (Table 3). The variables with *p-*values M < 0.1 in the univariate analysis underwent binary logistic multivariate analysis. After large artery atherosclerosis of stroke and the presence of diabetes were adjusted for, the results demonstrated that the baseline elevated SBP (OR = 1.213, 95% CI, 1.005–1.465, *p* = 0.045), baseline NIHSS (OR = 1.515, 95% CI, 1.186–1.935, *p* = 0.001), prior hyperlipemia (OR = 3.065, 95% CI, 1.107–8.482, *p* = 0.031), cardioembolic stroke (OR = 0.323, 95% CI, 0.120–0.871, *p* = 0.025), and END at 24 h (OR = 4.531, 95% CI, 1.950–10.533, *p* < 0.001) after IVT were independently associated with unfavorable outcomes at 3 months in mild AIS patients (Table 4). SICH could not be included in the logistic analysis due to the small number of patients.

**Table 3:** Characteristics of mild acute ischemic stroke patients with IVT with or without favorable outcomes at 3 months.

	**Favorable outcome** **at 3 months** *N* **= 176**	**Unfavorable outcome** **at 3 months** ***N* = 57**	***p*-value**
Age, IQR	66 (58–73)	69 (62–75)	0.107
Female, *N* (%)	61 (34.7)	23 (40.4)	0.433
ONT, (mean ± SD)	174.82 ± 80.27	193.04 ± 78.00	0.131
Baseline NIHSS, IQR	3 (2–4)	3 (2–5)	**0.001**
Baseline SBP, mmHg	147.99 ± 18.51	154.68 ± 17.27	**0.016**
Baseline DBP, mmHg	85.10 ± 11.18	83.61 ± 11.09	0.463
Smoking, *N* (%)	45 (26.9)	16 (28.1)	0.865
Hypertension, *N* (%)	118 (67.0)	42 (73.7)	0.413
Atrial fibrillation, *N* (%)	29 (16.5)	6 (10.5)	0.393
Coronary heart disease, *N* (%)	7 (4.0)	3 (5.3)	0.710
Diabetes, *N* (%)	34 (19.3)	18 (31.6)	**0.067**
Hyperlipemia, *N* (%)	13 (7.4)	9 (15.8)	**0.070**
Prior stroke or TIA, *N* (%)	27 (15.3)	13 (23.2)	0.222
TOAST criteria			
Large artery atherosclerosis, *N* (%)	41 (23.3)	23 (40.4)	**0.017**
Cardioembolic, *N* (%)	42 (23.9)	6 (10.5)	**0.037**
Lacunar, *N* (%)	60 (34.1)	19 (33.3)	1.000
Undetermined cause, *N* (%)	30 (17.0)	8 (14.0)	0.683
Other etiology, *N* (%)	3 1.7)	1 (1.8)	1.000
Hemorrhagic transformation, *N* (%)	6 (3.4)	3 (5.3)	0.692
HI-I, *N* (%)	5 (2.8)	0 (0)	
HI-II, *N* (%)	1 (0.6)	0 (0)	
PH-I, *N* (%)	0 (0)	1 (1.8)	
PH-II, *N* (%)	0 (0)	2 (3.5)	
Symptomatic intracerebral hemorrhage, *N* (%)	0 (0)	2 (3.5)	0.059
END at 24 h, *N* (%)	14 (8.0)	17 (29.8)	** <0.001**
Length of stay in hospital, IQR, day	8 (7–12)	12 (9–17)	** <0.001**

*SBP, systolic blood pressure; DBP, diastolic blood pressure; HI-I, hemorrhagic infarction type; HI-II, hemorrhagic infarction type II; PH-I, parenchymal hemorrhage type I; PH-II, parenchymal hemorrhage type II*.

**Table 4:** Binary logistic analysis of risk factors of functional outcome at 3 months after IVT.

	**OR**	**95% CI**	***p***
Baseline SBP	1.213	1.005–1.465	**0.045**
Baseline NIHSS	1.515	1.186–1.935	**0.001**
Diabetes	1.551	0.728–3.305	0.256
Hyperlipemia	3.065	1.107–8.482	**0.031**
Large artery atherosclerosis	1.543	0.737–3.231	0.250
Cardioembolic	0.323	0.120–0.871	**0.025**
END at 24 h	4.531	1.950–10.533	** <0.001**

## Discussion

In this study, we demonstrated that an elevated baseline SBP and coronary heart disease were independently associated with END. In addition, an elevated baseline SBP, baseline NIHSS, prior hyperlipemia, cardioembolic stroke, and END at 24 h after IVT were useful for predicting an unfavorable outcome in mild AIS patients.

An elevated baseline SBP was an independent predictor of END and unfavorable outcomes at 3 months after IVT in mild AIS patients. After the occurrence of ischemic stroke, SBP might remain elevated to maintain constant cerebral perfusion (17). Current guidelines (12) recommend that blood pressure should be controlled (<180/105 mmHg) in the first 24 h after IVT. However, the relationship between blood pressure and functional outcome exhibits a U-shape (18, 19). Both high SBP and low SBP are correlated with unfavorable outcomes in ischemic stroke patients (18, 20, 21). On one hand, lower SBP might lead to unfavorable outcome by reducing cerebral hemodynamic reserve and cerebral hypoperfusion (22). On the other hand, higher SBP might be associated with unfavorable outcome due to cerebral edema, stroke recurrence, and hemorrhagic transformation (18). Yan et al. found that when the SBP was maintained within a range of 140–149 mmHg for the first 24 h after IVT, neurological deterioration was the lowest (19). In our study, the mean SBP in non-END patients was 148.47 ± 18.15 mmHg, which fell within the recommended 140–149 mmHg range.

The mechanism by which SBP contributes to END is unclear. Yan He et al. found that blood pressure was directly proportional to serum levels of MMP-9 and AQP-4 at 24 h after thrombolysis (19). Thus, they presumed that the SBP that occurred with END might be associated with oxidative stress-induced blood–brain barrier disruption and AQP-4 upregulation (19). Also, high SBP could increase the risk of cerebral edema, hemorrhagic transformation, and stroke recurrence, which are associated with unfavorable outcomes in ischemic stroke patients. We observed that END in mild AIS patients directly impacted the unfavorable outcome at 3 months after IVT. This result was consistent with a prior study (23).

Coronary heart disease is defined as the patient having a history of acute coronary syndrome or angina pectoralis. It is well known that ischemic stroke and coronary heart diseases have the same risk factors. It has been reported that coronary heart disease is correlated with an unfavorable outcome in AIS patients (24). Our study demonstrated that coronary heart disease was an independent predictor of END after IVT in mild AIS patients, but the underlying mechanism is unknown. In fact, there was no difference in ejection fraction of coronary heart disease patients between the END group and non-END group (66.00 ± 7.83% vs. 65.17 ± 4.54%, *p* = 0.967). It is notable that we observed 10 patients with coronary heart disease that included four (12.9%) in the END group and six (3.0%) in the non-END group. In the END group, three of the four coronary heart disease patients concurrently experienced cerebral vascular stenosis. Conversely, no patient experienced cerebral vascular stenosis in the other group. Therefore, we proposed that coronary heart disease associated with END was possibly related to the fact that the patients with coronary heart disease also had a high occurrence of cerebral vascular stenosis. This possibility could be confirmed in a future study.

In this study, the incidence of hemorrhagic transformation was 3.9% (9/233), and SICH was 0.9% (2/233). We found that hemorrhagic transformation did not affect the occurrence of an unfavorable outcome at 3 months after IVT in mild AIS patients. Although two SICH patients experienced an unfavorable outcome at 3 months, the incidence of SICH in mild AIS patients with IVT was not higher than other ischemic stroke patients with IVT. Previous studies demonstrated that IVT did not increase the risk of SICH in mild ischemic stroke patients (25).

Baseline NIHSS has been determined to be an independent predictor of unfavorable outcomes in mild ischemic stroke after IVT (26, 27), and the result in this study was the same as in these prior studies. For cardioembolic stroke, we found those patients had higher rate of favorable outcomes at 3 months after IVT. Similar studies were published (28, 29). It seemed that IVT was more effective in cardioembolic stroke. This result might be associated with the composition of the thrombus. The thrombus of cardioembolic stroke contains more fibrin and platelet, but other thrombi contain more red blood cells (28). Meanwhile, rt-PA has high banding affinity for fibrin and might be more prone to result in thrombus dissolution (28).

We also observed that hyperlipemia was associated with unfavorable outcomes in mild AIS patients. Hypertriglyceridemia and hypercholesterolemia were both included in hyperlipemia. A previous study confirmed that hypertriglyceridemia might be a predictor of END (30). However, high LDC-C early in the course of stroke has been associated with a favorable outcome at 3 months in mild ischemic stroke (31). The specific pathophysiological mechanism underlying this association remains unclear. Because of our retrospective design, we did not subdivide the patients with hypertriglyceridemia and hypercholesterolemia.

Our study is mainly limited by its retrospective design of single center. First, although we collected data using an established prospective stroke registry, a risk of selection bias is possible. Second, we found baseline SBP, baseline NIHSS, a history of prior hyperlipemia, cardioembolic stroke, and END at 24 h after IVT were associated with unfavorable outcome at 3 months in mild stroke patients. But our registry did not collect the outcome data of mild ischemic stroke patients without IVT. There might be other factors associated with unfavorable outcome of mild ischemic stroke patients without IVT, which will be collected and studied in our future work. Third, we found that coronary heart disease had a high occurrence of cerebral vascular stenosis and proposed that coronary heart disease associated with END was possibly related to this fact. But we have no complete data on large vessel stenosis of all included mild stroke patients. We could not get a conclusion on in this study on whether mild stroke patients with large vessel stenosis were more likely to experience END. This possibility could be confirmed in a future study that uses a larger sample size.

## Conclusion

In this study, 24.5% of patients with mild ischemic stroke experienced an unfavorable outcome after IVT. The incidence of SICH was low (0.9%). Moreover, elevated baseline SBP was an independent predictor of END at 24 h and an unfavorable outcome at 3 months after IVT. Thus, blood pressure might be rigorously controlled for mild ischemic stroke patients during and after IVT. This conclusion needs to be confirmed with a larger sample size and the inclusion of additional blood pressure-related parameters in future work.

## Data Availability Statement

The original contributions presented in the study are included in the article/supplementary material, further inquiries can be directed to the corresponding author.

## Ethics Statement

The studies involving human participants were reviewed and approved by the Human Ethics Committee of Shaoxing People's Hospital. The patients/participants provided their written informed consent to participate in this study.

## Author Contributions

HT was responsible for setting this topic, designing protocol, statistical analysis, and writing papers. SY was responsible for designing protocol and statistical analysis. CW was responsible for data collection and assessing hemorrhagic transformation. YZ was responsible for data collection and follow-up work. All authors contributed to the article and approved the submitted version.

## Conflict of Interest

The authors declare that the research was conducted in the absence of any commercial or financial relationships that could be construed as a potential conflict of interest.

## Publisher's Note

All claims expressed in this article are solely those of the authors and do not necessarily represent those of their affiliated organizations, or those of the publisher, the editors and the reviewers. Any product that may be evaluated in this article, or claim that may be made by its manufacturer, is not guaranteed or endorsed by the publisher.
